# High expression of myoferlin is associated with poor outcome in oropharyngeal squamous cell carcinoma patients and is inversely associated with HPV-status

**DOI:** 10.18632/oncotarget.7625

**Published:** 2016-02-23

**Authors:** Bhavna Kumar, Nicole V. Brown, Benjamin J. Swanson, Alessandra C. Schmitt, Matthew Old, Enver Ozer, Amit Agrawal, David E. Schuller, Theodoros N. Teknos, Pawan Kumar

**Affiliations:** ^1^ Department of Otolaryngology-Head and Neck Surgery, The Ohio State University, Columbus, OH 43210, USA; ^2^ Comprehensive Cancer Center, The Ohio State University, Columbus, OH 43210, USA; ^3^ Center for Biostatistics, The Ohio State University, Columbus, OH 43210, USA; ^4^ Department of Pathology, The Ohio State University, Columbus, OH 43210, USA; ^5^ Current affiliation: Department of Pathology and Laboratory Medicine, Emory University School of Medicine, Atlanta, GA 30303, USA

**Keywords:** myoferlin, OPSCC, HPV, IL-6, nanog

## Abstract

Myoferlin (MYOF) is a member of ferlin family of membrane proteins that was originally discovered as a muscle specific protein. Recent studies have shown that myoferlin is also expressed in other cell types including endothelial cells and cancer cells. However, very little is known about the expression and biological role of myoferlin in head and neck cancer. In this study, we examined expression profile of myoferlin in oropharyngeal squamous cell carcinoma (OPSCC) and assessed its correlation with disease progression and patient outcome. In univariate analyses, nuclear MYOF was associated with poor overall survival (p<0.001) and these patients had 5.5 times increased hazard of death (95% Cl 3.4-8.8). Nuclear myoferlin expression was also directly associated with tumor recurrence (p<0.001), perineural invasion (p=0.008), extracapsular spread (p=0.009), higher T-stage (p=0.0015) and distant metastasis (p<0.001). In addition, nuclear MYOF expression was directly associated with IL-6 (p<0.001) and inversely with HPV status (p=0.0014). In a subgroup survival analysis, MYOF nuclear+/IL-6+ group had worst survival (84.6% mortality), whereas MYOF nuclear-/IL-6- had the best survival. Similarly, patients with HPV-negative/MYOF-positive tumors had worse survival compared to HPV-positive/MYOF-negative. Taken together, our results demonstrate for the first time that nuclear myoferlin expression independently predicts poor clinical outcome in OPSCC patients.

## INTRODUCTION

Head and neck squamous cell carcinoma (HNSCC) is the eighth most frequent cancer worldwide and includes the following subsites: oral cavity, nasopharynx, oropharynx, hypopharynx and larynx [1, 2]. The majority of patients present with locally advanced stage disease and require multimodality therapy with surgery followed by chemotherapy/radiotherapy or organ preserving chemotherapy/radiotherapy [3-5]. These treatments are intensive and associated with severe acute toxicity, such as mucositis, dermatitis and dysphagia as well as long term sensorineural hearing loss, permanent xerostomia and altered swallowing function [6, 7]. Although advancements in the anti-cancer treatments including surgery, radiation and chemotherapy have increased the local control of HNSCC, the overall survival rates have not improved significantly over the last three decades [8]. Five year survival rates for patients with early stage localized head and neck cancers are more than 80%, but drop to 40% when the disease has spread to the neck nodes, and to below 20% for patients with distant metastatic disease [8-10]. Patients with head and neck cancer encompass a heterogeneous group and can be further subdivided into two distinct tumor subtypes; human papillomavirus (HPV)-negative and HPV-positive tumors [11]. Majority of HNSCC patients with HPV-positive tumors respond very well to traditional chemo-radiotherapy and demonstrate significantly favorable clinical outcomes [12, 13]. However, there is a small subset of HPV-positive patients that do not respond well to standard therapy and show markedly poor clinical outcome [14, 15]. In contrast to HPV-positive patients, the majority of HPV-negative patients are usually smokers, have more aggressive disease and many of these patients develop resistance to chemotherapy leading to poor prognosis [13]. Therefore, it is becoming increasing clear that treatment for patients with HNSCC has to shift from a single disease approach to tailoring the therapy based on patient's tumor characteristics.

Recently, we made a novel discovery in our laboratory that a muscle-specific protein, myoferlin, is markedly upregulated in HNSCC. Myoferlin, a member of ferlin family of proteins, was originally discovered as a candidate gene for muscular dystrophy and cardiomyopathy [16]. The ferlin family is named for its homology to the *Caenorhabditis elegans* protein Fer-1 [17, 18]. Humans have six Fer-1-like genes that form the ferlin family: dysferlin (Fer1L1), otoferlin (Fer1L2), myoferlin (Fer1L3), Fer1L4, Fer1L5, and Fer1L6 [19-24]. The ferlins share similar domain architecture: a carboxy-terminal transmembrane domain and multiple amino-terminal C2 domains [22, 25]. The ferlin proteins harbor the capacity to bind directly to negatively charged phospholipids and additionally scaffold a number of distinct proteins via their C2 domains. The ferlin family of proteins has been implicated in fusion events in muscle, including myoblast fusion and vesicle trafficking [26-29]. Dysferlin, otoferlin and myoferlin have been extensively studied in muscle cells and it has been found that they predominantly maintain plasma membrane integrity [30, 31]. Myoferlin is a 230-kDa protein that is highly expressed in myoblasts, especially those myoblasts that have begun to differentiate [16, 26, 31]. Recent studies have shown that in addition to muscle cells, myoferlin is also expressed in endothelial and cancer cells [32-37]. In cancer cells, myoferlin overexpression have been shown either at mRNA or protein levels using cancer cell lines or small number of patient tumor samples [33, 35-37]. Difilippantonio S et al, used suppression subtractive hybridization (SSH) technique to identify differentially expressed genes in lung squamous cell carcinoma as compared to normal bronchial epithelial cells [36]. They showed that myoferlin is significantly upregulated in lung cancer cells. Abba MC et al, showed enhanced expression of myoferlin in breast cancer cells using Serial Analysis of Gene Expression (SAGE) technique [37]. Turtoi A et al, used a proteomic approach to identify potentially accessible proteins overexpressed in pancreas ductal adenocarcinoma (PDAC) and found that myoferlin is markedly upregulated in PDAC cells [35]. Recently, Turtoi A et al, also showed a significantly higher myoferlin expression in breast adenocarcinoma as compared to normal breast tissue using both proteomic approaches and immunohistochemistry (IHC) [33]. However, there is no reported study that has examined myoferlin expression in a large cohort of patient population and its correlation with patient outcome.

In this study, we examined the expression and localization of myoferlin in OPSCC tumors and correlated it with patient survival, HPV status, IL-6 expression, nanog expression and other clinical and pathological variables. Our results show a direct correlation between myoferlin expression and poor overall survival and an inverse correlation with HPV status. Interestingly, we observed that nuclear myoferlin expression was highly predictive of poor overall survival and was directly associated with high IL-6 and nanog expression. In addition, nuclear myoferlin was directly associated with tumor recurrence, perineural invasion, extracapsular spread (ECS), higher T-stage and distant metastasis.

## RESULTS

**Figure 1:**
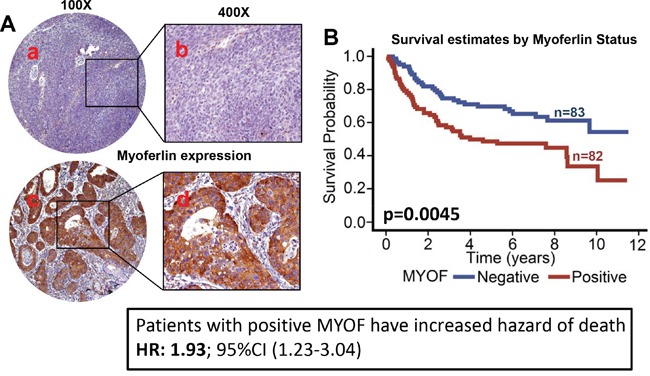
Myoferlin expression is associated with poor overall survival in OPSCC Tissue microarrays (TMAs) containing primary tumor samples from OPSCC patients were stained for myoferlin expression. **A.** Representative pictures of tumor cores negative for myoferlin expression (a and b) and positive for myoferlin expression (c and d). **B.** Overall survival estimates of patients according to myoferlin expression.

To evaluate the expression pattern and clinical importance of myoferlin, IL-6 and nanog in HNSCC, we assessed the expression of these biomarkers in TMA's constructed using 211 surgically treated oropharyngeal squamous cell carcinoma samples. Patient characteristics are listed in Table 1. Representative images of staining for myoferlin, IL-6, nuclear myoferlin and nanog are included in Figures 1, 3, 4 and 5, respectively. 117 tumors (55.7%) were HPV16 positive and 93 tumors (44.3%) were negative. The median follow-up time was 5.5 years (range 0.1-11.5). The 5 year survival rates for the whole group were 60.6%, 78.6% for the HPV-positive group and 37.6% for the HPV-negative group.

**Table 1:** Patient Demographics and Clinical Characteristics

Patient Characteristics	n	%
**Age (years), mean (SD)**	57.7	9.7
**Marital Status**		
Single/Divorced/Widowed	87	45.6
Married	104	54.5
**Race**		
African American/Black	9	4.3
White	202	95.7
**Sex**		
Female	45	21.3
Male	166	78.7
**Smoking Status**		
≤10 pack years	51	25.1
>10 pack years	152	74.9
**Extracapsular Spread**		
No	118	57.8
Yes	86	42.2
**HPV16 Status**		
Negative	93	44.3
Positive	117	55.7
**Mucosal Margins**		
Free of Carcinoma	174	83.7
Positive Margins	34	16.4
**Node Stage**		
N0	28	13.3
N1	48	22.8
N2	126	59.7
N3	9	4.3
**Perineural Invasion**		
No	157	74.8
Yes	53	25.2
**TNM Stage**		
I	5	2.4
II	11	5.2
IIII	51	24.2
IV	144	68.3
**Tumor Stage**		
T1	45	21.3
T2	86	40.8
T3	41	19.4
T4	39	18.5

**Figure 2:**
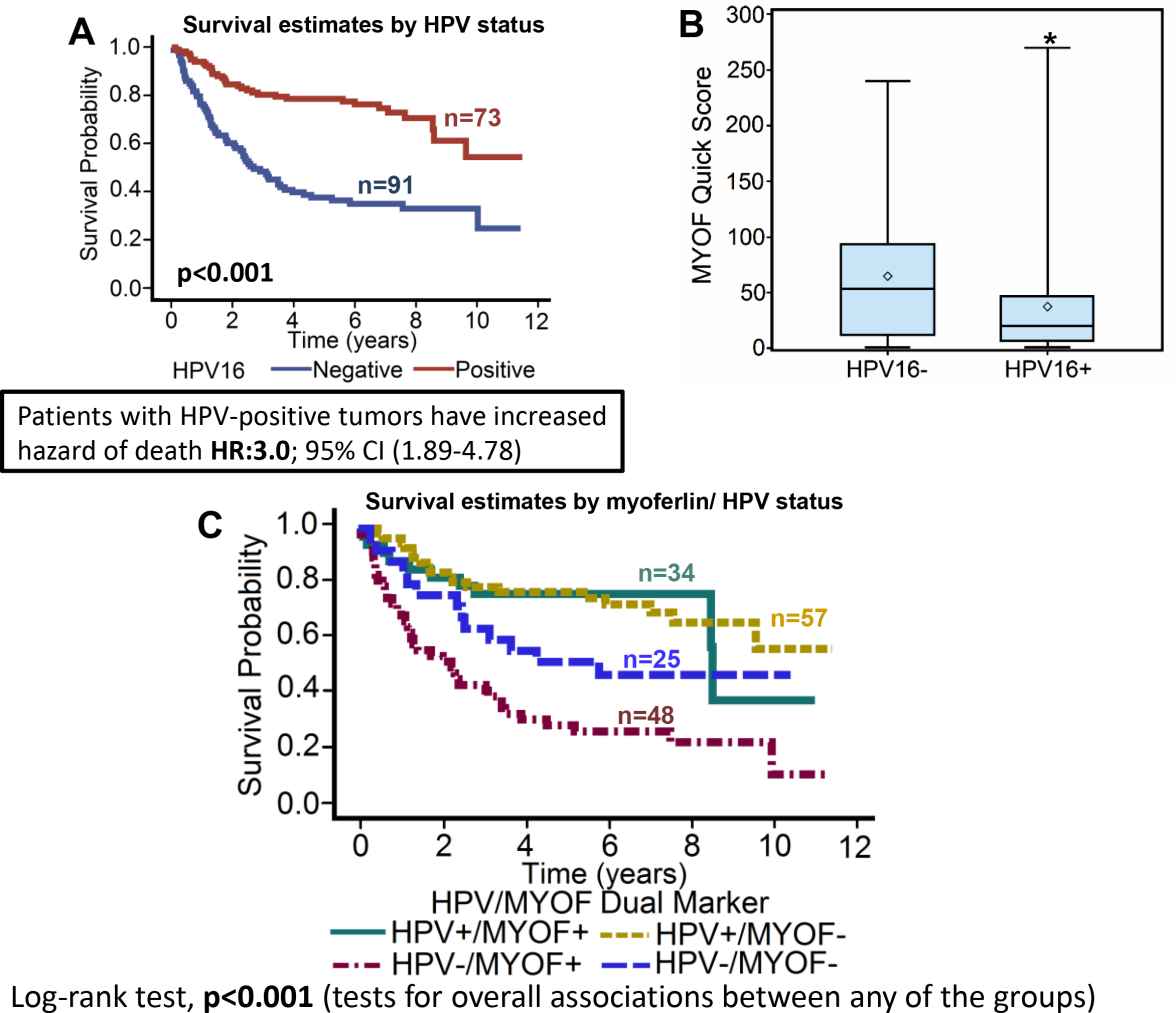
Myoferlin expression is significantly lower in HPV-positive tumors as compared to HPV-negative tumors **A.** Overall survival estimates of patients according to HPV16 status. **B.** Myoferlin expression in HPV16-positive versus HPV16-negative patients. *, represent a significant difference in myoferlin expression in HPV16-positive (HPV16+) tumor samples as compared to HPV16-negative (HPV16-) tumor samples. **C.** Survival estimates of patients according to myoferlin expression and HPV status.

**Figure 3:**
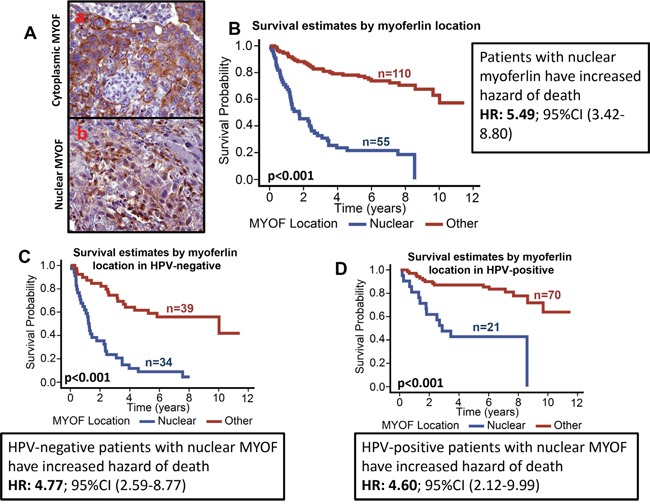
Patients with nuclear myoferlin have significantly poor survival **A.** Representative pictures of membranous/cytoplasmic myoferlin (a) and nuclear myoferlin staining (b). **B.** Overall survival estimates of patients according to myoferlin location. **C.** Overall survival estimates according to myoferlin location in HPV-negative patients. **D.** Overall survival estimates according to myoferlin location in HPV-positive patients.

**Figure 4:**
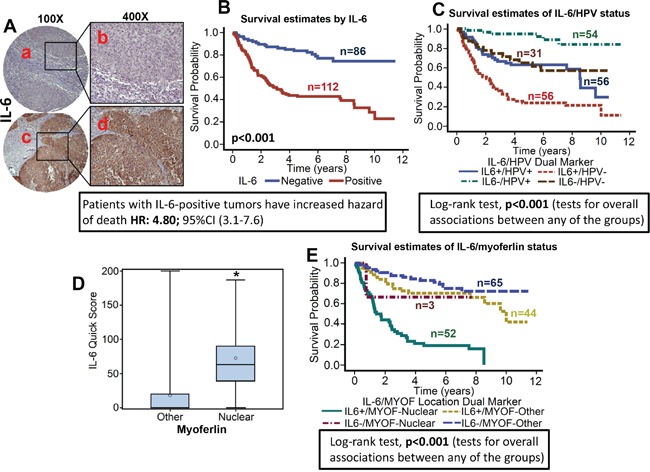
High IL-6 expression is associated with poor overall survival and directly correlates with nuclear myoferlin expression TMAs were stained for IL-6 expression. **A.** Representative pictures of tumor cores negative for IL-6 expression (a and b) and positive for IL-6 expression (c and d). **B.** Overall survival estimates of patients according to IL-6 expression. **C.** Survival estimates of patients according to IL-6 expression and HPV status. **D.** IL-6 levels in tumor samples with nuclear or non-nuclear (other) myoferlin expression. **E.** Overall survival estimates according to myoferlin location and IL-6 expression.

**Figure 5:**
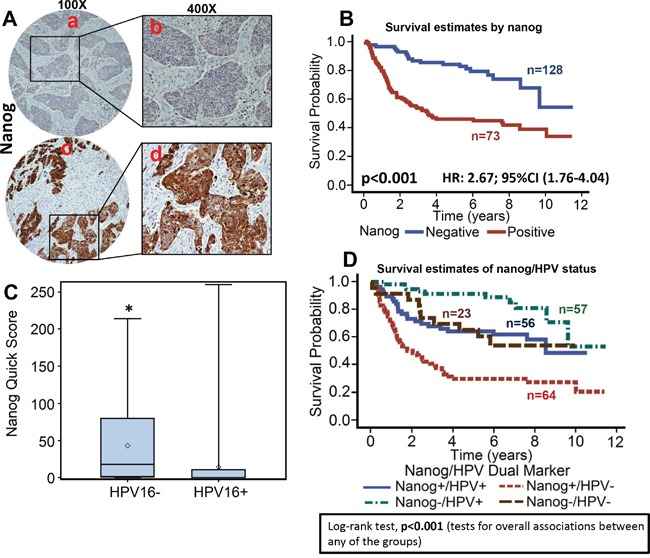
High nanog expression is associated with poor overall survival TMAs were stained for nanog expression. **A.** Representative pictures of tumor cores negative for nanog expression (a and b) and positive for nanog expression (c and d). **B.** Overall survival estimates of patients according to nanog expression. **C.** Nanog expression in HPV-negative and HPV-positive tumor samples. *, represent a significant difference in nanog expression in HPV-positive tumor samples as compared to HPV-negative tumor samples. **D.** Overall survival estimates according to nanog expression and HPV status.

### Myoferlin expression is directly associated with poor overall survival and inversely associated with HPV status

Myoferlin expression was evaluable in 165 tumors and was found to be overexpressed in 82 (49.7%) tumors (Figure 1A). When analyzed as a continuous variable, increasing expression of myoferlin was significantly associated with worse overall survival (stain proportion p=0.0068, quick score p=0.0045). Patients with myoferlin positive tumors had significantly higher hazard of death (HR: 1.9; 95% CI 1.2-3.0) as compared to patients that were negative for myoferlin expression (Figure 1B). We and others have previously shown that OPSCC patients with HPV-positive tumors have superior outcomes compared to HPV-negative patients [12, 13]. In this present study, we also observed that patients with HPV-negative tumors have significantly higher hazard of death (HR: 3.0; 95% CI 1.9-4.8) as compared to patients with HPV-positive tumors (Figure 2A). In addition, HPV-negative tumors had significantly higher expression of myoferlin as compared to HPV-positive tumors (Figure 2B, p=0.0014). In the subgroup survival analysis, HPV-negative/myoferlin-positive (HPV-/MYOF+) group had the worst survival compared to the HPV-positive/myoferlin-negative (HPV+/MYOF−) (Figure 2C, p<0.001). Overall association between different groups is presented in Table 2.

**Table 2:** Log-rank test for overall association between different groups

Multiple Comparisons for the Log-rank Test		
Strata Comparison		p-value
HPV+/MYOF+	HPV+/MYOF−	1.000
HPV+/MYOF+	HPV−/MYOF+	<.001
HPV+/MYOF+	HPV−/MYOF−	0.723
HPV+/MYOF−	HPV−/MYOF+	<.001
HPV+/MYOF−	HPV−/MYOF−	0.160
HPV−/MYOF+	HPV−/MYOF−	0.001

Significant differences between
HPV+/MYOF+ and HPV−/MYOF+ HPV+/MYOF− and HPV−/MYOF+ HPV−/MYOF+ and HPV−/MYOF−

### Nuclear myoferlin expression is highly predictive of poor clinical outcome

Myoferlin expression was predominately cytoplasmic in the OPSCC tumor samples. However, we also observed nuclear myoferlin expression in 55/165 (33%) tumors. Tumors that expressed myoferlin in the nucleus were categorized as nuclear positive. Those tumors that had cytoplasmic or plasma membrane expression were classified as other. Higher nuclear myoferlin expression was associated with worse overall survival (p<0.001). Patients whose tumors were nuclear myoferlin positive had a 5.5 hazard ratio of death (95% CI: 3.4-8.8) as compared to those in which myoferlin was not present in the nucleus (Figure 3A-3B). A larger proportion of patients whose tumors were HPV-negative had nuclear myoferlin expression as compared to HPV-negative with non-nuclear myoferlin expression (61.8% versus 35.8%; p=0.0015). Similarly, larger proportion of patients with nuclear myoferlin expression had T3/T4 tumors (54.6% versus 29.1%; p=0.0015), had perineural invasion (40.7% versus 20.9%; p=0.008), had extracapsular spread (59.6% versus 37.6%; p=0.009), had a recurrence (50% versus 18.3%; p<0.001), and distant metastasis (45.4% versus 0.0%; p<0.001) as compared to those with non-nuclear myoferlin expression. In subgroup survival analysis, nuclear myoferlin expression was highly predictive of poor overall survival in both HPV-negative (Figure 3C, HR 4.8; 95% CI: 2.6-8.8) and HPV-positive (Figure 3D, HR 4.6; 95% CI: 2.1-9.9) patients.

### IL-6 overexpression is associated with poor overall survival and is directly correlated with nuclear myoferlin expression

IL-6 expression was evaluable in 198 tumors and was found to be overexpressed in 112 (56.6%) tumors (Figure 4A). When analyzed as a continuous variable, increasing expression of IL-6 was significantly associated with worse overall survival of patients with OPSCC (stain proportion p<0.001, stain intensity p<0.001, quick score p<0.001). The hazard of death for patients with positive IL-6 expression was 4.9 (95% CI: 3.1-7.6, Figure 4B). In the subgroup survival analysis, IL-6+/HPV- group had the worst survival as compared to the other three groups (Fisher's exact test, p<0.001, Figure 4C). Interestingly, nuclear myoferlin expression was directly associated with high IL-6 expression in the primary tumor samples (p<0.001; Figure 4D). In the subgroup survival analysis, IL-6+/MYOF-nuclear group had the worst survival (84.6% mortality) as compared to the other three groups (Fisher's exact test, p<0.001, Figure 4E). In addition, IL-6 overexpression was also directly associated with tumor recurrence (p<0.001), perineural invasion (p=0.005), extracapsular spread (p=0.0107) and inversely associated with HPV status (p=0.0153).

### Nanog overexpression is associated with poor overall survival and is directed correlated with IL-6 and nuclear myoferlin expression

Nanog expression was evaluable in 201 OPSCC tumors (Figure 5A). When analyzed as a continuous variable, increasing expression of nanog was significantly associated with worse overall survival (stain proportion p<0.001; stain intensity p<0.001; quick score p=0.0026). The hazard of death for patients with positive nanog expression was 2.7 (95% CI: 1.7-4.0, Figure 5B). Patients whose tumors were HPV-negative had higher expression of nanog (p<.001, Figure 5C). In the subgroup survival analysis, nanog+/HPV- group had the worst survival as compared to the other three groups (Fisher's exact test, p<0.001, Figure 5D). In addition, high nanog expression was directly associated with recurrence (p<0.001), smoking status (>10pk-year, p<0.001), perineural invasion (p=0.037), and N stage (N2/N3, p=0.03).

**Figure 6:**
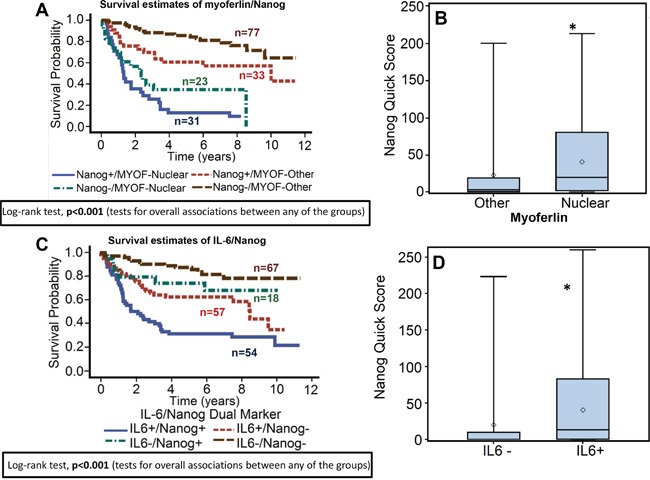
Nanog expression is directly correlated with IL-6 and nuclear myoferlin expression **A.** Overall survival estimates according to nanog and myoferlin expression. **B.** Nanog expression in tumor samples with nuclear or non-nuclear (other) myoferlin expression. *, represent a significant difference in nanog expression in tumor samples with nuclear myoferlin expression as compared to tumor samples with non-nuclear myoferlin. **C.** Overall survival estimates according to nanog and IL-6 expression. **D.** Nanog expression in IL-6-negative or positive tumor samples. *, represent a significant difference in nanog expression in IL-6-positive (IL-6+) tumor samples as compared to IL-6-negative (IL-6-) tumor samples.

When looking at the correlation between the expressions of the 3 biomarkers, we also found that as IL-6 expression increases, so does the expression of nuclear myoferlin and nanog (Table 3). We also found that tumors with nuclear myoferlin expression had higher IL-6 (p<0.001, Figure 4D), and nanog expression (p<0.001, Figure 6B). In the subgroup survival analysis, nanog+/MYOF-nuclear group (Figure 6A) and IL-6+/nanog+ group (Figure 6C) had the worst survival as compared to the other groups (Fisher's exact test, p<0.001). In addition, nanog expression was also directly associated with IL-6 expression (p<0.001, Figure 6D).

**Table 3:** Correlation between IL-6 expression, myoferlin location and nanog expression

IL-6 Quick Score						
Variable	N	Minimum	25th Pctl	Median	75th Pctl	Maximum
Myoferlin Location						
Other	109	0.00	0.00	0.00	20.00	200.00
Nuclear	55	0.00	40.00	63.33	90.00	186.67
Nanog						
Negative	124	0.00	0.00	0.00	34.17	170.00
Positive	72	0.00	0.83	46.67	74.17	200.00

### Multivariate analysis

After adjusting for other covariates, myoferlin location, tumor stage and HPV status were significantly associated with overall survival in a multivariable model (Table 4).

**Table 4:** Overall survival analysis in a multivariable model

Variables	Level	HR	95% CI		p-value
Myoferlin Location	Nuclear	3.456	1.997	5.982	<.001
Tumor Stage	T3/T4	2.081	1.303	3.324	0.002
Node Stage	N2/N3	1.270	0.778	2.073	0.338
Smoking Status (Pack Years)	> 10 pack years	1.266	0.685	2.336	0.451
HPV16	Negative	2.054	1.219	3.461	0.006
IL-6	Positive	1.800	0.936	3.460	0.078
Nanog	Positive	1.186	0.683	2.059	0.544

## DISCUSSION

In this study, we examined the expression of myoferlin in surgically treated oropharyngeal squamous cell carcinoma samples and correlated that with survival, HPV status, and other clinical and pathological variables. For the first time, we show that myoferlin expression, particularly nuclear myoferlin expression, is highly predictive of poor overall survival, tumor recurrence, perineural invasion, higher T stage and distal metastasis in OPSCC patients. Most of the published studies have examined the role of myoferlin in plasma membrane stability or growth factor receptor recycling. The results from this study suggest that myoferlin might play an important role in the nucleus.

Over the past decade, it has become apparent that the incidence of “classic” tobacco/alcohol-induced HNSCC has declined, but at the same time, HNSCC caused by HPV has risen sharply [38]. HPV16 is the most prevalent subtype and it accounts for ≈90% of HPV-related HNSCC [39, 40]. Intriguingly, patients with HPV-related HNSCC tend to have far better prognosis than HPV-negative counterparts [12, 13]. A number of potential models have been proposed to explain this clinical outcome disparity between HPV-positive versus HPV-negative patients. However, we still know very little about the precise molecular mechanism(s) that could explain this clinical outcome disparity. In this study, we show an inverse correlation between HPV-positivity and myoferlin levels. These results suggest that differential myoferlin expression might be one of the contributing factors for this prognosis disparity in HPV-positive versus HPV-negative patients. It is possible that HPV-positive tumors lack the oncogenic hit(s) that upregulate myoferlin expression or HPV oncogenes (E6/E7) actively downregulate myoferlin expression. Additional mechanistic studies are underway in our laboratory to answer these questions. Although the majority of HNSCC patients with HPV-positive tumors have better outcome as compared to HPV-negative tumors, we and others have recently described a small subgroup of HPV-positive patients that have very aggressive tumors that do not respond to standard therapy leading to poor clinical outcome [14, 41-42]. Our results from this study show that myoferlin particularly nuclear myoferlin expression could be used as a prognostic marker to distinguish HPV-positive patients that have poor clinical outcome and tailor their treatment regimen accordingly.

We had recently shown that IL-6 overexpression promotes tumor metastasis in head and neck cancer [43]. In addition, IL-6 overexpressing cells (CAL27-IL-6) were resistant to cisplatin treatment. While characterizing these cells for myoferlin expression, we made an intriguing discovery that total myoferlin expression was similar in IL-6^high^ and IL-6^low^ expressing cells, but there was markedly altered subcellular localization of myoferlin. Myoferlin was predominantly localized in the nucleus of IL-6 overexpressing cells, whereas it was predominantly localized in cytosol/membrane of parental cells (IL-6^low^). In this study, we have examined if similar to our *in vitro* studies, myoferlin was present in nucleus and was there any clinical significance of altered subcellular distribution of myoferlin. Our results show that myoferlin was present in the nucleus in 33% of the tumors and patients whose tumors were nuclear myoferlin positive had 5.5 times the hazard of death than patients whose tumors had cytosolic/membranous myoferlin expression. In addition, nuclear myoferlin expression was directly associated with high IL-6 expression in the primary tumor samples thereby suggesting that IL-6 might be involved in promoting the nuclear translocation of myoferlin. We are currently testing this hypothesis in a mechanistic study. A number of studies have shown that IL-6 modulate cancer stem cell phenotype by regulating nanog expression, [44-46]. In this study, we also show that high nanog expression is associated with poor overall survival and nanog expression is directly correlated with IL-6 and nuclear myoferlin expression. This study therefore suggests that nuclear myoferlin could be used as a potential prognostic marker for poor clinical outcome and to individualize treatment strategies for HNSCC patients. Additional studies are required to further validate the use of nuclear myoferlin in the prognosis of HNSCC patients.

## MATERIALS AND METHODS

### Study population

Oropharyngeal squamous cell carcinoma tissue specimens were obtained from surgical resections of patients at The Ohio State University James Cancer Hospital and Solove Research Institute between 2002 to 2009. All patients underwent surgical resection as a first line of therapy with a curative intent. This was followed by no additional treatment or adjuvant chemotherapy and/or radiotherapy as needed. The Ohio State University Institutional review Board approved a retrospective analysis study of these specimens and a waiver of HIPAA authorization was obtained. Patient characteristics, including age, race, gender, marital status, smoking status, pathological variables including tumor size, nodal status, AJCC stage, extracapsular spread, perineural invasion and clinical variables including survival and recurrence outcomes were recorded. Recurrence was defined as biopsy or radiographically confirmed occurrence of a new suspicious mass in the region of the resected primary tumor or in any other sites within the first 5 years of surgical resection.

### Tissue microarray (TMA)

Paraffin-embedded archival tissue blocks and their matching H&E-stained slides were retrieved from the Department of Pathology. A pathologist marked the areas with cancer and adjacent normal on the H&E slides. Representative regions (three cores of tumor tissue and one core of adjacent normal tissue) were sampled using a 0.6-mm punch on a master TMA blocks. The TMA's were constructed by the Histology Core in the Department of Pathology. Unstained sections were cut and used for immunohistochemical staining.

### Immunohistochemistry and scoring

TMA slides were stained to assess the tumor expression of IL-6, Myoferlin and Nanog using immunohistochemistry as previously described [13]. Briefly, slides were deparaffinzed in xylene, and rehydrated in decreasing concentrations of ethyl alcohol. Antigen retrieval was performed in a decloaking chamber (Biocare Medical, LLC, Concord, CA, USA) using antigen unmasking buffer (Dako) for 20 minutes at 120°C. After a 20 minutes cool down period at room temperature, sections were incubated with dual endogenous enzyme block (Dako) for 10 minutes at room temperature. Non-specific binding sites were blocked by incubating with PBS/serum from species in which the secondary antibody was raised. Sections were then incubated with primary antibody (goat anti-IL-6: R&D Systems, 4°C overnight; rabbit anti-myoferlin: Prestige antibody, Sigma, 1 hour at room temperature; rabbit anti-Nanog: Epitomics/Abcam, 1 hour at room temperature). After washes, slides were incubated with biotinylated donkey anti-goat (Jackson Immunoresearch; for IL-6) or, biotinylated anti-rabbit (Vectastain Elite Kit; for Myoferlin and Nanog) for 30 minutes at room temperature. Earls Buffered Salt Solution containing 0.1% saponin was used as wash buffer for IL-6 IHC. PBS was used for Myoferlin and Nanog IHC. Slides were rinsed in wash buffer and incubated with avidin-biotin complex (Vector Laboratories, Burlingame, CA, USA) for 30 minutes. They were then rinsed in wash buffer and incubated with 3,3′-diaminobenzidine (Sigma). The slides were rinsed in water, counterstained with Mayer's hematoxylin, mounted and coverslipped. Stained slides were interpreted by a pathologist who was blinded to treatment outcome at the time of review. Tumor cells were scored for stain proportion (0-100%) and intensity (1: none, 2: low, 3: moderate, 4: high). A quick score was generated by multiplying the stain proportion scores with stain intensity to obtain values between 0-400.

In-situ hybridization (GenPoint, Dako) was used for the detection of HPV16 in the tumor specimens as previously described [14]. The tumors were categorized as positive when specific nuclear stain was observed in the tumor cell nuclei.

### Statistical analyses

Overall survival was defined as time from the date of surgery to date of death, with patients alive at the date of last observation censored. Cox proportional hazards models were used to assess univariate associations of biomarkers as predictors for death. Unadjusted hazard ratios (HR) and 95% confidence intervals (CI) are reported. A multivariable model including nuclear myoferlin status, tumor stage, node stage, smoking status (based on pack years), HPV16 status, IL-6 expression, and nanog expression was built to estimate adjusted HRs. To assess dual marker interactions, comparisons of survival curves were evaluated using the log-rank test with adjustment for multiple comparisons made by Bonferonni corrections. Mann-Whitney tests were used to assess associations between biomarkers, demographic, or clinical characteristics and IL-6, myoferlin, or nanog expression. Fisher's exact or Chi-square tests were used, as appropriate, to assess associations between categorical variables. All analyses were conducted in SAS, version 9.3 (SAS Institute, Cary, North Carolina).
